# Expression of hormone receptors is associated with specific immunological profiles of the breast cancer microenvironment

**DOI:** 10.1186/s13058-023-01606-7

**Published:** 2023-01-31

**Authors:** Toru Hanamura, Shigehisa Kitano, Hiroshi Kagamu, Makiko Yamashita, Mayako Terao, Takuho Okamura, Nobue Kumaki, Katsuto Hozumi, Takayuki Iwamoto, Chikako Honda, Sasagu Kurozumi, Naoki Niikura

**Affiliations:** 1 Department of Breast Oncology, Tokai University School of Medicine, 143 Shimokasuya, Isehara-shi, Kanagawa Prefecture 259-1193 Japan; 2 Division of Cancer Immunotherapy Development, Center for Advanced Medical Development, The Cancer Institute Hospital of JFCR, 3-8-31, Ariake, Koto, Tokyo 135-8550 Japan; 3 Division of Respiratory Medicine, Saitama Medical University International Medical Center, 1397-1, Yamane, Hidaka-shi, Saitama Prefecture 350-1298 Japan; 4 Department of Pathology, School of Medicine, Tokai University, 143 Shimokasuya, Isehara-shi, Kanagawa Prefecture 259-1193 Japan; 5 Department of Immunology, Tokai University School of Medicine, 143 Shimokasuya, Isehara-Shi, Kanagawa Prefecture 259-1193 Japan; 6 Breast and Endocrine Surgery, Okayama University Hospital, 2-5-1 Shikata-cho, Kitaku, Okayama Prefecture 700-8558 Japan; 7 Department of General Surgical Science, Gunma University Graduate School of Medicine, 39-22, Showa-machi 3-chome, Maebashi-shi, Gunma Prefecture 371-8511 Japan; 8 Department of Breast Surgery, International University of Health and Welfare, 4-3, Kozunomori, Narita-shi, Chiba Prefecture 286-8686 Japan

**Keywords:** Breast cancer, Estrogen receptor, Progesterone receptor, Androgen receptor, Tumor immunity, Microenvironment, Immune cell composition

## Abstract

**Background:**

Elucidating the unique immunoregulatory mechanisms in breast cancer microenvironment may help develop new therapeutic strategies. Some studies have suggested that hormone receptors also have immune regulatory functions, but their mechanisms are not fully understood. In this study, we have comprehensively analyzed the relationship between the expressions of estrogen (ER), progesterone (PgR), and androgen receptors (AR), and the immunological profile in breast cancer.

**Methods:**

Using publicly available gene expression profile datasets, METABRIC and SCAN-B, the associations between the expressions of hormone receptors and the immune cell compositions in breast cancer tissue, estimated by CIBERSORTx algorithm, were analyzed. We histologically evaluated tumor-infiltrating lymphocytes (hTIL), PD-L1 (hPD-L1) expression, and the infiltration of 11 types of immune cells by flow cytometry (FCM) for 45 breast cancer tissue samples. The relationships between them and the expressions of ER, PgR, and AR of tumor tissues, evaluated immunohistochemically, were analyzed.

**Results:**

Expressions of *ESR1*, *PGR*, and *AR* were negatively correlated with overall immune composition. Expressions of ER and AR, but not that of PgR, were inversely associated with hTIL and hPD-L1 expression. FCM analysis showed that the expressions of ER and AR, but not that of PgR, were associated with decreased total leukocyte infiltration. Both CIBERSORTx and FCM analysis showed that ER expression was associated with reduced infiltration of macrophages and CD4+ T cells and that of AR with reduced macrophage infiltration.

**Conclusion:**

Hormone receptor expression correlates with specific immunological profiles in the breast cancer microenvironment both at the gene and protein expression levels.

**Supplementary Information:**

The online version contains supplementary material available at 10.1186/s13058-023-01606-7.

## Background

Breast cancer is the most commonly occurring cancer in women. Despite recent advances in multimodal treatment, advanced and recurrent cases are challenging to cure [1], and the urgent need to develop innovative treatment strategies has to be addressed. Owing to the development of immune checkpoint inhibitors (ICIs) that reinvigorate the adaptive immune response in the tumor microenvironment and the successful application of these ICIs in various neoplasms, tumor immunology has recently gained renewed interest across multiple cancers [2, 3]. ICIs have also been applied for breast cancer treatment, but their effectiveness is limited, likely due to the immunosuppressive tumor microenvironment [4–9]. Elucidating the unique immunomodulatory mechanisms of the breast cancer microenvironment will provide significant insights into the development of new therapeutic strategies.

Hormone dependency is one of the prominent biological features of breast cancer. Approximately 70% of breast cancer cells express the estrogen receptor (ER), resulting in ER-dependent growth of breast cancer [1]. Therefore, treatment strategies that inhibit ER function are frequently used as postoperative adjuvant therapy for patients with early-stage breast cancer and as systemic therapy for metastatic cases [1]. The progesterone receptor (PgR) is clinically considered a complementary marker of hormone dependency in breast cancer [1, 10] because it is driven partly, but not exclusively, by ER-mediated transcriptional events [11, 12]. PgR is a binding partner and major modifier of ER-mediated processes, suggesting its additional role in breast cancer other than its identification as an ER activity marker [13, 14]. The androgen receptor (AR) is a nuclear transcription factor with a diverse range of biological actions, mainly in the development and maintenance of the male reproductive system [15]. It is widely expressed in all breast cancer subtypes to varying extents, with approximately 60–80% of the cases being AR-positive [16–18]. Furthermore, according to The Human Protein Atlas, AR expression, at both gene and protein levels, is second-highest in breast cancer, after prostate cancer, among various malignancies [19]. Although the function of AR in breast cancer depends on the tumor subtype, treatment, and other factors, it is suggested to have a tumor-promoting role [20–22], thereby attracting attention as a new therapeutic target for breast cancer treatment [23, 24].

Some recent studies have suggested that sex steroid hormones and their receptor signaling have immune regulatory functions. In vitro studies have shown that estrogen can expand the regulatory T-cell fraction and reduce the function of antigen-presenting cells [25, 26]. In addition, estrogen can promote immune tolerance by interfering with human leukocyte antigen-II expression in ER-positive breast cancer cell lines [27]. These findings suggest that estrogen signaling in the tumor microenvironment regulates anti-tumor immunity [2]. In agreement with this, hormone receptor-positive breast cancer is characterized by low infiltration of tumor-infiltrating lymphocytes (TILs) and minimal response to ICIs [5, 9, 28–32]. To the best of our knowledge, the immunological function of PgR in breast cancer has not yet been reported. However, limited studies have shown a relationship between PgR expression and tumor immunity in breast cancer; PgR expression is inversely associated with programmed death-ligand 1 (PD-L1) expression in epithelial cells or the stroma and the infiltration of CD8+ T and CD20+ B cells [9, 33, 34]. Further, immune regulatory functions of AR signals have been demonstrated via in vivo models of various autoimmune diseases and some malignancies [35]. Moreover, in breast cancer, AR expression is inversely correlated with immune cell infiltration and cytotoxic immune activity, suggesting an immunosuppressive effect of AR signals [36–38].

Despite fragmentary evidence on sex steroid hormone signals and tumor immunity, the interactions between hormone receptors and immune cells are not well understood because of the complexity of the immune milieu in the breast cancer microenvironment and limited reports on systematic evaluation of immune cell composition in breast cancer tissue [39]. In this study, we systematically analyzed the relationship between the expression of sex steroid hormone receptors such as ER, PgR, and AR and the immunological profiles of breast cancer tissues. Our results demonstrated that hormone receptor expression, at both gene and protein levels, correlates with specific immunological profiles of the breast cancer microenvironment, strongly suggesting their direct or indirect immunomodulatory role.

## Materials and methods

### Gene expression profile datasets

Two publicly available gene expression profile datasets of patients with breast cancer used in this study were the Molecular Taxonomy of Breast Cancer International Consortium (METABRIC) [40, 41] cohort (*n* = 1904) and the Sweden Cancerome Analysis Network-Breast (SCAN-B) [42, 43] cohort (*n* = 3273). Gene expression data of METABRIC and SCAN-B, generated by microarray and RNA-sequencing, were downloaded from the cBioPortal: https://www.cbioportal.org/ (accessed on 20/2/2019) and Gene Expression Omnibus: https://www.ncbi.nlm.nih.gov/geo/ (accessed on 16/6/2019), respectively. The inclusion criteria and clinicopathological information for each cohort have been provided in the original papers. The METABRIC cohort included patients with primary invasive breast cancer, including < 1% metastatic breast cancer. However, information on the use of preoperative chemotherapy was not provided. On the contrary, the SCAN-B cohort consisted of patients with non-metastatic primary invasive breast cancer and included the cases of preoperative chemotherapy. Both cohorts included all breast cancer subtypes, and all samples were obtained from primary lesions.

### CIBERSORTx

Immune cell composition in breast cancer tissue was determined from gene expression profiles using a bioinformatics algorithm, CIBERSORTx: https://cibersortx.stanford.edu/ (accessed on 25/2/2022) [44]. Briefly, “LM22” representing the profiling of 22 functionally defined human immune cell types [45] was applied as a signature matrix file. The non-log-transformed gene expression data from METABRIC and SCAN-B were applied to the mixture file. The program was run in the absolute mode with 100 permutations. A B-mode batch correction was applied, and quantile normalization was set as disabled. Absolute scores representing the overall immune content and absolute abundance of each immune cell fraction in the mixture were produced by the algorithm. Cases with a CIBERSORTx *p*-value < 0.05 were filtered and selected for subsequent analysis. Absolute scores and absolute abundance of each cell type in a mixture were used for correlation analysis with the expression values of the indicated genes that were log2-transformed.

### Gene set enrichment analysis (GSEA)

The gene expression values in the METABRIC dataset were log2-transformed before performing GSEA [46, 47]. Hallmark gene set collections (50 gene sets) representing specific well-defined biological states or processes were obtained from MsigDB v7.1: https://www.gsea-msigdb.org/gsea/msigdb/ (accessed on 13/5/2020) and applied to GSEA using GSEA software v4.0: https://www.gsea-msigdb.org/gsea/msigdb/ (accessed on 21/11/2019). While performing GSEA, the permutations were set at 1000 with phenotype as the permutation type. Expression values of the indicated genes were used as phenotype labels, and Pearson’s correlation was set as the metric for ranking genes. Thresholds for nominal *p*-value and false discovery rate (FDR) *q*-value were set at < 0.05 and < 0.25, respectively. According to user guidelines, the SCAN-B dataset was not applied to GSEA because of the incompatible normalization method used for this analysis.

### Patients

In a previous study, immune cell composition of breast cancer tissue was evaluated using flow cytometry (FCM) and an association between histologically assessed expression of TIL and PD-L1 and the immunological profile of the tumor microenvironment was reported [39]. The inclusion criteria and patient characteristics have also been described previously [39], and this dataset was used for further analyses. Briefly, 47 breast cancer samples were obtained, regardless of clinicopathological factors or treatment histories, except for patients with distant metastases or complete clinical responses to neoadjuvant chemotherapy. None of the patients had received irradiation or endocrine therapy before surgery. Clinicopathological data were collected by reviewing the case records. Two cases were excluded from the analysis, and the reasons are mentioned in section "TIL preparation/ FCM analysis".

### Histological evaluation of hormone receptors and tumor immunity-related biomarkers

Rabbit monoclonal antibodies for ER (SP1) and PgR (1E2) were purchased from Ventana Medical Systems Japan Inc. (Tokyo, Japan) and for AR (AR27) from Leica Biosystems Inc. (Wetzlar, Germany). The immunohistochemistry (IHC) staining was performed using the VENTANA BenchMark ULTRA automated IHC device (Roche Diagnostics, Basel, Switzerland) for ER and PgR and BOND-III automated IHC device (Leica Biosystems Inc.; Wetzlar, Germany) for AR. The antigen–antibody complex was visualized using diaminobenzidine and counterstained with hematoxylin. The nuclear staining of ER, PgR, and AR in carcinoma cells was counted, and the percentage of immunoreactive cells was determined. ER and PgR were determined as positive when nuclear staining-positive cells were ≥ 10%. [48]. However, the accepted cutoff value for AR expression is not known; since the median value of nuclear staining-positive cells for AR was 60% in the present study, AR was considered positive at ≥ 60%. We diverted the data from histological analysis of the expression of TIL and PD-L1 from our previous study, referred to as hTIL and hPD-L1, respectively [39]. According to the International TILs Working Group guidelines [49], the percentages of TILs in stromal tissue sections stained with hematoxylin and eosin (H&E) were evaluated and categorized into three grades: low (0–10%), intermediate (10–40%), and high (40–90%). PD-L1 expression was assessed by IHC using an anti-PD-L1 antibody (SP142; Spring Bioscience, Pleasanton, CA, USA). Tumors with ≥ 1% immune cells showing cytoplasmic and/or membrane PD-L1 staining were determined to be PD-L1 positive [50]. A previous study report accounted that in a case, hTIL and hPD-L1 could not be evaluated because the tumor tissue was insufficient for analysis.

### TIL preparation/ FCM analysis

FCM data from breast cancer tissue samples were obtained from a previously reported study, which contains the detailed method [39]. To perform the analysis, fresh breast cancer tissues were mechanically dissociated and filtered using a 70-micron cell strainer. From the filtered cell suspension, mononuclear cell components were separated by density-gradient centrifugation and subjected to FCM analysis. Samples stained with an antibody cocktail were detected using LSR II Fortessa with the fluorescence-activated cell sorting Diva software (BD Biosciences). All analyses were performed using the FlowJo software v10.6.1 (BD Biosciences). The list of antibodies used, gating strategy, and definitions of immune cell fractions have been described in the previous report [39]. According to the staining profile of the surface antigen, evaluated by FCM, the cells were classified as follows: leukocytes, total T cells (total T), CD4+ T cells (CD4+ T), CD8+ T cells (CD8+ T), B cells (B), monocytes/macrophages (Mo/Mφ), non-classical monocytes (CD16+ Mo), myeloid-derived suppressor cells (MDSCs), dendritic cells (DCs), myeloid dendritic cells (mDCs), natural killer (NK) cells, minor NK cells, and natural killer T cells (NKT). As mentioned in section "Patients", two cases were excluded because they had a low number of living cells (count < 1000) in the FCM analysis of tumor tissue. Leukocyte density was defined as the count of total CD45+ cells per weight of tumor tissue (count/g), as described in a previous paper [51]. Similarly, we determined the count of each immune cell fraction per weight of the tissue fragment (count/g).

### Statistical analyses

GraphPad Prism ver. 9.1.0 software was used for statistical analyses and graph preparation. The gene expression and FCM data were tested using the D'Agostino–Pearson normality test, which showed non-normal distribution in almost all the datasets. Correlation analyses between groups were performed using Spearman’s rank correlation coefficient. |*r*-value|> 0.3 and a significant *p*-value was defined as a positive or negative correlation [52, 53]. The Mann–Whitney U test and Fisher’s exact test were used to compare continuous and categorical variables between the two unpaired groups, respectively. The *p*-value < 0.05 was defined as statistically significant. In the previous study [39], the FCM data contained outliers. Here, although all analyses were performed without omitting outliers, we identified the outliers using the robust regression and outlier removal method, excluded them, and performed all statistical analyses to ensure the reliability of our analyses. Statistics with omitted outliers are shown in each figure along with the original data.

## Results

### Gene expression levels of hormone receptors in breast cancer tissue

**Fig. 1:**
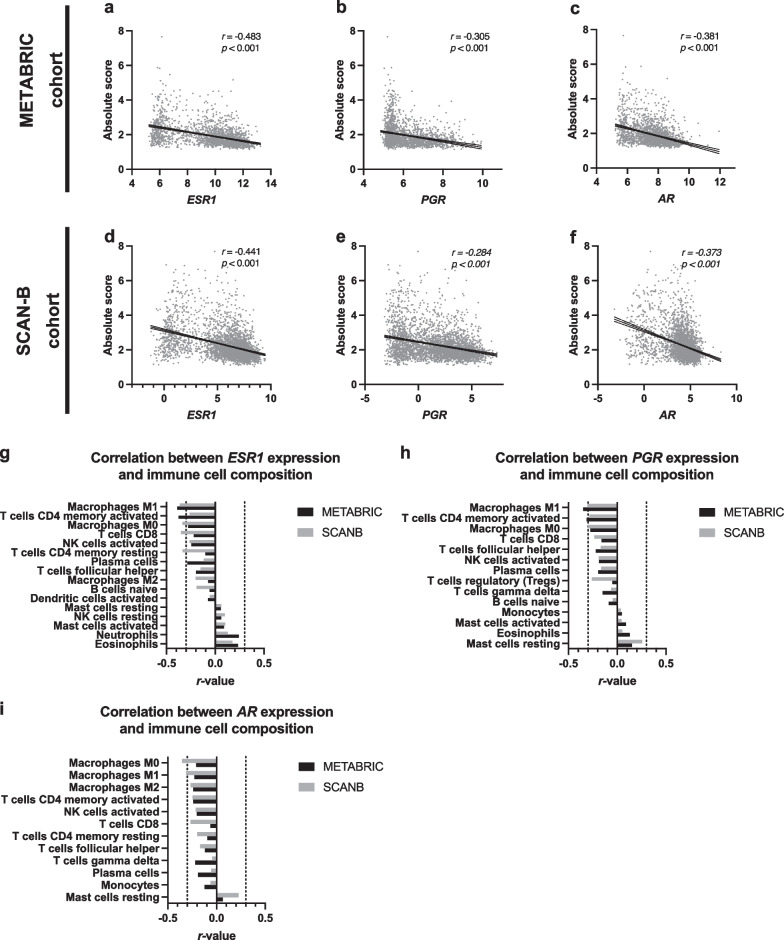
Association between gene expression levels of hormone receptors and immunological profile of the breast cancer tissue. **a**–**f** Scatterplots showing a correlation between the gene expression levels of estrogen receptor 1 (*ESR1*), progesterone receptor (*PGR*), and androgen receptor (*AR*), and absolute score estimated by CIBERSORTx. **g**–**i** Graphs showing the correlation coefficient (*r*-value) between indicated gene expression and the absolute amount of various immune cell fractions estimated by CIBERSORTx. Immune cell fraction data showing consistently significant *p*-values in the METABRIC and SCAN-B datasets have been displayed in ascending order of *r*-value

Gene expression of estrogen receptor 1 (*ESR1*), *PGR*, and *AR* negatively correlated with the absolute score in the METABRIC cohort (*r* > − 0.3, *p* < 0.05); however, the negative correlation was weak for *PGR* in the SCAN-B cohort (*r* = − 0.284, *p* < 0.05) (Fig. 1a–f). *ESR1* expression levels inversely correlated with Mφ M1 and M0, CD4 + T (memory activated), CD8+ T, and CD4+ T (memory resting) in at least one dataset (*r* < − 3, *p* < 0.05) (Fig. 1g). Likewise, *PGR* expression levels inversely correlated with Mφ M1 and M0 and CD4+ T (memory activated) in at least one dataset (*r* < − 3, *p* < 0.05) (Fig. 1h). Similarly, *AR* expression levels inversely correlated with Mφ M0 and M1 in at least one dataset (*r* < − 3, *p* < 0.05) (Fig. 1i). These results suggest that the expression of hormone receptors *ESR1*, *PGR*, and *AR* is inversely associated with the total immune content and the infiltration of specific immune cell fractions in the tumor tissue.

### Correlation of histologically assessed TIL and expression of PD-L1 with protein expression of ER and AR

**Fig. 2:**
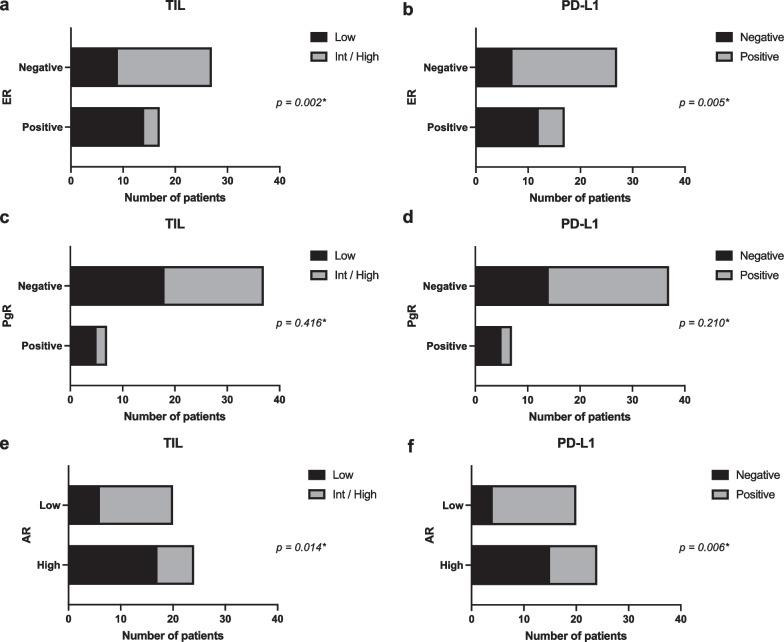
Histological assessment of the expression of tumor-infiltrating lymphocytes (TILs) and programmed death-ligand 1(PD-L1) based on protein expression status of estrogen receptor (ER) and androgen receptor (AR). **a**–**f** Graphs showing the number of patients with positive or negative immune-related markers according to hormone receptor status. Fisher’s exact test was used to compare categorical variables between two groups; actual *p*-values are shown in the figure

The status of ER, PgR, AR, PD-L1 (hPD-L1), and TIL (hTIL) in breast cancer tissue was histologically evaluated by IHC and H&E staining using the samples obtained at our facility from 45 patients with breast cancer. A summary of clinicopathological findings according to hormone receptor status is shown in Additional file 1: Tables S1–S3. The correlation between hormone receptor expression and immune-related biomarkers was further analyzed at the protein level. Expression of both hTIL and hPD-L1 showed a negative correlation with the protein expression of ER and AR, but not with that of PgR, probably due to the small number of PgR-positive cases (Fig. 2a–f).

### Association of hormone receptor status with leukocyte density and tumor-infiltrating immune cells in breast cancer tissue

**Fig. 3:**
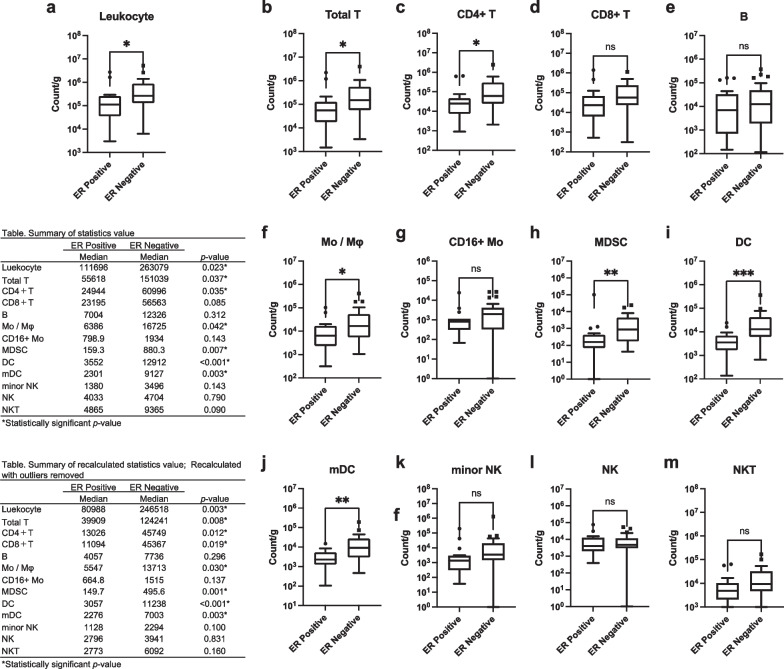
Association between estrogen receptor (ER) status and subsets of tumor-infiltrating immune cells. **a** Total leukocyte density (count/g) in breast cancer tissue according to ER status. **b**–**m** Count of each immune cell fraction per unit weight of the tissue (count/g) according to ER status. A tabulated summary of the statistics values and recalculated statistics values excluding outliers is shown on the upper and lower left sides of the figure, respectively. ns *p* > 0.05 (not significant); *p < 0.05; **p < 0.01; ***p < 0.001

**Fig. 4:**
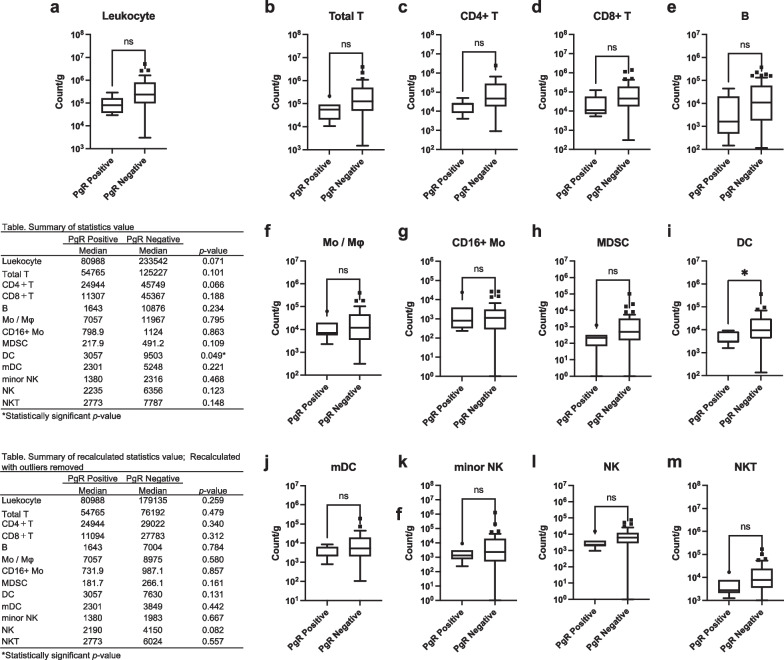
Association between progesterone receptor (PgR) status and subsets of tumor-infiltrating immune cells. **a** Total leukocyte density (count/g) in breast cancer tissue according to PgR status. **b**–**m** Count of each immune cell fraction per unit weight of the tissue (count/g) according to PgR status. A tabulated summary of the statistics values and recalculated statistics values excluding outliers is shown on the upper and lower left sides of the figure, respectively. ns *p* > 0.05 (not significant); *p < 0.05; **p < 0.01; ***p < 0.001

**Fig. 5:**
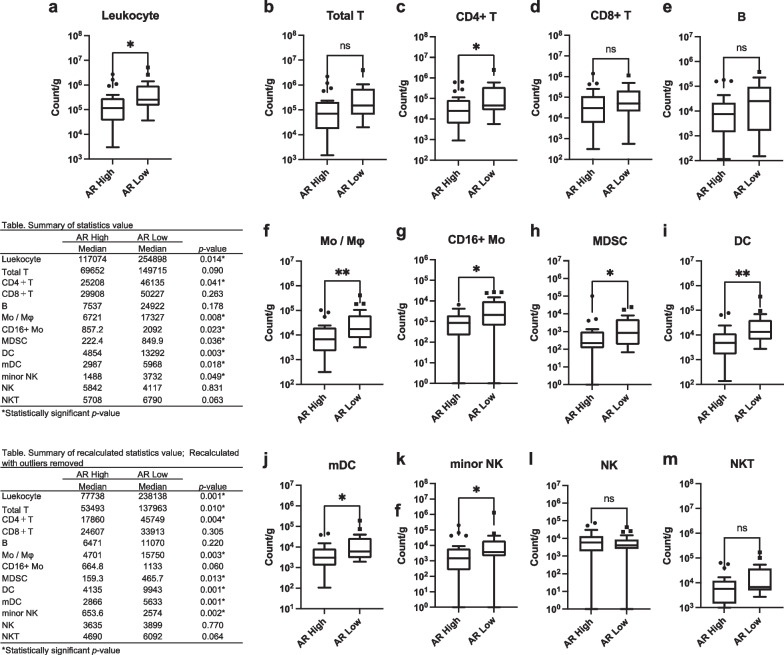
Association between androgen receptor (AR) status and subsets of tumor-infiltrating immune cells. **a** Total leukocyte density (count/g) in breast cancer tissue according to AR status. **b**–**m** Count of each immune cell fraction per unit weight of the tissue (count/g) according to AR status. A tabulated summary of the statistics values and recalculated statistics values excluding outliers is shown on the upper and lower left sides of the figure, respectively. ns *p* > 0.05 (not significant); *p < 0.05; **p < 0.01; ***p < 0.001

The relationship between each hormone receptor status, density of leukocytes, and each immune cell fraction in breast cancer tissue was analyzed. ER positivity was associated with decreased leukocyte density and reduced infiltration of total T, CD4+ T, Mo/Mφ, MDSC, DC, and mDC in breast cancer tissue (Fig. 3a–m). PgR positivity was associated with decreased infiltration of DC but not with leukocyte density or other immune cell fractions (Fig. 4a–m). AR positivity was associated with decreased leukocyte density and infiltration of CD4+ T, Mo/Mφ, CD16+ Mo, MDSC, DC, mDC, and minor NK cells (Fig. 5a–m). These results suggest a strong association of the expression of ER and AR with decreased infiltration of leukocytes and specific immune cell fractions such as CD4+ T and Mo/Mφ into breast cancer tissue.

## Discussion

This study systematically analyzed the relationship between hormone receptor expression (at both gene and protein levels) and the immunological profile of breast cancer tissues, including multiple immune cell fractions. Expression of ER and AR in breast cancer tissues was associated with a decreased infiltration of immune cells into the tumor microenvironment. More specifically, ER expression was associated with decreased infiltration of macrophages and CD4+ T cells, while AR expression with reduced macrophage infiltration. These results were consistent at the gene and protein expression levels.

The present analysis showed that gene expression of hormone receptors was inversely correlated to total immune cell infiltration into the tumor microenvironment (Fig. 1a–f). This was further verified by performing GSEA on the METABRIC dataset, which analyzed the relationship between the expression levels of hormone receptors and hallmark gene set collection (50 gene sets) representing specific well-defined biological states or processes. Expression levels of *ESR1*, *PGR,* and *AR* showed a significant positive correlation with gene sets representing estrogen response, such as estrogen response early and estrogen response late (Additional file 1: Figure S1a–c). On the other hand, *ESR1* and *AR* expression levels showed a significant negative correlation with multiple gene sets representing immunological processes such as inflammatory response, allograft rejection, complement, and interferon-gamma response (Additional file 1: Figure S1d, f). Likewise, a significant negative correlation was observed between *PgR* expression level and gene sets of inflammatory response, allograft rejection, and interferon-gamma response (Additional file 1: Figure S1e). These findings suggest that the gene expression levels of hormone receptors, such as *ESR1*, *PGR,* and *AR,* correlate with immunosuppressive phenotypes of breast cancer.

Several preclinical studies have suggested that ER signaling may suppress the immune response of breast cancer [2, 25–27], and ER-positive breast cancers have shown reduced TIL infiltration and minimal response to ICI [5, 9, 28–32]. This is consistent with the present findings, suggesting an immunomodulatory function of ER. Although numerous studies have analyzed the relationship between a single immune cell lineage and hormone receptor expression [32, 54–57], few reports have systematically investigated the multiple immune cell composition of breast cancer tissues [51, 58–60]. The present study reports the first systematic analysis demonstrating that ER expression is preferentially associated with reduced infiltration of macrophages and CD4+ T cells (Fig. 1g, 3c, 3f). This result is consistent with earlier reports that show a negative correlation between ER expression and intratumoral infiltration of macrophages and CD4+ T cell [54–57]. Further, an inverse correlation between ER expression and CD8+ T cells has been reported previously [32, 54]; however, in our analysis, this correlation was weaker than that for CD4+ T cells, both at the gene and protein levels (Figs. 1g and 3). In addition, *PGR* expression showed a weaker correlation with total immune content than *ESR1* and *AR*, as estimated by CIBERSORTx. Moreover, the correlation between PgR status and hTIL and hPD-L1 was insignificant at the protein level, probably due to the small number of PgR-positive samples. To validate these findings, a greater number of samples are required to be analyzed.

AR signaling is suggested to have immunomodulatory functions in some malignancies, including breast cancer [35–38]; however, its immunological role in breast cancer has not been fully validated. To the best of our knowledge, a single study has investigated the relationship between immune cell composition and AR expression in breast cancer using the CIBERSORT algorithm [38]. Tumors with high AR expression were reported to be associated with pro-cancer regulatory T cells, and those with low AR expression were associated with anti-cancer immune cells, such as CD4, CD8, gamma delta T cells, and memory B cells in ER-positive breast cancer. In our study, CIBERSORTx was run in absolute mode and included all subtypes in the analysis; therefore, a simple comparison was not possible. High AR-expressing tumors showed reduced macrophage infiltration into the tumor microenvironment; this finding was consistent with CIBERSORTx and FCM analysis results.

In the present study, expressions of ER and AR were inversely correlated with hPD-L1 expression (Fig. 2b, f). We analyzed the PD-L1 expression in each immune cell fraction by FCM analysis, similar to our previous study [39]. According to hormone receptor status, we found that ER positivity was associated with decreased PD-L1 expression in Mo/Mφ and mDC (Additional file 1: Figure S2d, h), PgR positivity with decreased PD-L1 expression in MDSCs and NK cells (Additional file 1: Figure S3f, j), and AR positivity with decreased PD-L1 expression in CD8+ T and Mo/Mφ cells (Additional file 1: Figure S4b, d). PD-L1 expression reflects ongoing (or active) immune responses in addition to immunosuppression via the PD-1/PD-L1 pathway [60–62]. Thus, ER positivity and AR positivity may reflect decreased immune response in the breast cancer microenvironment.

Findings of this study suggest that the hormone receptor signals may primarily affect specific immune cell lineages. In particular, the expression of ER and AR showed a negative correlation not only with total immune content in the tumor microenvironment but also with certain immune cell subsets such as macrophages and CD4+ T cells. This indicates that specific immune cell lineages may be primary targets of the immunoregulatory function of the hormone receptor signals. Our next goal is to verify this hypothesis using an in vitro or in vivo analysis model.

In our previous study [39], a relatively small number of samples were used for FCM analysis, and compared to those of the general breast cancer cohort, the clinicopathological characteristics of tumors were biased with larger invasive tumor sizes, more ER-negative cases, and higher Ki67 cases. Similarly, in this study, the small sample size prevented us from performing subgroup analyses based on the tumor subtype. Therefore, the inclusion of a greater number of samples and more detailed analyses are required in future studies. In this exploratory analysis, the cut-off value for ER and PgR positivity was set at ≥ 10% according to our previous study [39]. However, in a recent clinical guideline update [63], 1% is recommended as a cutoff value for ER- and PgR-positive cells because of limited but present data on endocrine therapy benefit for cancers with 1% to 10% of cells staining ER positive. Therefore, the analysis of Figs. 2, 3, and 4 was repeated using the cutoff points 1% for ER and PgR, as shown in Additional file 1: Figure S5a–h. When the cutoff value is set to 1%, for Fig. 2, the expression of hPD-L1 showed a negative correlation with the protein expression of PgR (Fig S5d). Similarly, for Fig. 3, the significant association between ER positivity and some immune cell compositions were lost (i.e., leukocyte density, infiltration of total T, CD4+ T, Mo/Mφ) (Fig S5e). For Fig. 4, PgR positivity gained significant association with decreased leukocyte density and reduced infiltration of total T, CD4+ T, MDSC, DC (Fig S5f). No meaningful changes were observed in other results. Only 2 cases each had changes in ER and PgR status due to changes in the cutoff values. Therefore, we speculate that the discrepancies in the analysis results are a consequence of the small sample size.

## Conclusions

In the present study, the gene expression levels of hormone receptors correlated with immunosuppressive phenotypes of breast cancer. The expression level of ER and AR proteins was associated with decreased tumor-infiltrating immune cells and decreased PD-L1 expression. These data suggest that hormone receptor signaling may suppress tumor immunity through a specific mechanism. Additionally, our data showed that certain immune cell lineages might get more strongly affected by hormonal signals than others, providing useful suggestions for further analysis of the effects of hormonal signals on tumor immunity.

## Supplementary Information

**Additional file 1: Fig. S1.** Association of typical biological processes with the expression of hormone receptors estimated using the gene expression profile of breast cancer. (a-f) Summary of results of GSEA performed on the METABRIC datasets is shown. Biological processes positively or inversely correlated with each gene expression are shown in the descending order of the absolute value of the normalized enrichment score (NSE) with absolute values of the log-transformed nominal p-values and FDR q-values. Thresholds of the nominal p-value and FDR q-value were set to < 0.05 and < 0.25, respectively, and the boundaries are shown in the graph by dashed lines. **Fig. S2.** Programmed death-ligand 1 (PD-L1) positive ratio in each immune cell fraction according to estrogen receptor (ER) status. (a-k) the percentage of PD-L1 positive cells in each immune cell fraction by ER status is shown. A summary of statistics value is shown at left side of figure. A summary of the recalculated statistics values excluding outliers is shown in the lower left of the figure. **Fig. S3.** Programmed death-ligand 1 PD-L1 positive ratio in each immune cell fraction according to progesterone receptor (PgR) status. (a-k) the percentage of PD-L1 positive cells in each immune cell fraction by PgR status is shown. A summary of statistics value is shown at left side of figure. A summary of the recalculated statistics values excluding outliers is shown in the lower left of the figure. **Fig. S4.** Programmed death-ligand 1 (PD-L1) positive ratio in each immune cell fraction according to androgen receptor (AR) status. (a-k) the percentage of PD-L1 positive cells in each immune cell fraction by AR status is shown. A summary of statistics value is shown at left side of figure. A summary of the recalculated statistics values excluding outliers is shown in the lower left of the figure. **Fig. S5.** Reanalysis of Figs. 2–4 using 1% cut-off point for ER and PgR. (a-d) Correlation of hTIL and hPD-L1 with protein expression of ER and PgR were reanalyzed using the cut-off points 1% for ER and PgR. (e, f) Association between ER / PgR status and subsets of tumor-infiltrating immune cells were reanalyzed using the cut-off points 1% for ER and PgR. (g, h) Recalculated statistics values excluding outliers are shown for ER and PgR status. **Table S1.** Clinical-pathological characteristics by ER status. **Table S2.** Clinical-pathological characteristics by PgR status. **Table S3.** Clinical-pathological characteristics by AR status.

## Data Availability

The datasets generated and/or analyzed during the current study are available upon reasonable request from the corresponding author.

## Abbreviations

ER: Estrogen receptor
PgR: Progesterone receptors
AR: Androgen receptor
hTIL: Histologically evaluated tumor-infiltrating lymphocytes
hPD-L1: Histologically evaluated programmed death ligand 1
FCM: Flow cytometry
ICIs: Immune checkpoint inhibitors
METABRIC: Molecular Taxonomy of Breast Cancer International Consortium
SCAN-B: Sweden Cancerome Analysis Network-Breast
GSEA: Gene Set Enrichment Analysis
IHC: Immunohistochemistry
H&E: Hematoxylin and eosin
Mo/Mφ: Monocytes/macrophages
CD16+ Mo: Non-classical monocytes
MDSCs: Myeloid-derived suppressor cells
DCs: Dendritic cells
mDCs: Myeloid dendritic cells
NK: Natural killer cells
NKT: Natural killer T cells
