# Correction: Expression of hormone receptors is associated with specific immunological profiles of the breast cancer microenvironment

**DOI:** 10.1186/s13058-023-01637-0

**Published:** 2023-03-20

**Authors:** Toru Hanamura, Shigehisa Kitano, Hiroshi Kagamu, Makiko Yamashita, Mayako Terao, Takuho Okamura, Nobue Kumaki, Katsuto Hozumi, Takayuki Iwamoto, Chikako Honda, Sasagu Kurozumi, Naoki Niikura

**Affiliations:** 1 Department of Breast Oncology, Tokai University School of Medicine, 143 Shimokasuya, Isehara-shi, Kanagawa Prefecture 259-1193 Japan; 2 Division of Cancer Immunotherapy Development, Center for Advanced Medical Development, The Cancer Institute Hospital of JFCR, 3-8-31, Ariake, Koto, Tokyo 135-8550 Japan; 3 Division of Respiratory Medicine, Saitama Medical University International Medical Center, 1397-1, Yamane, Hidaka-shi, Saitama Prefecture 350-1298 Japan; 4 Department of Pathology, School of Medicine, Tokai University, 143 Shimokasuya, Isehara-shi, Kanagawa Prefecture 259-1193 Japan; 5 Department of Immunology, Tokai University School of Medicine, 143 Shimokasuya, Isehara-shi, Kanagawa Prefecture 259-1193 Japan; 6 Breast and Endocrine Surgery, Okayama University Hospital, 2-5-1 Shikata-cho, Kitaku, Okayama Prefecture 700-8558 Japan; 7 Department of General Surgical Science, Gunma University Graduate School of Medicine, 39-22, Showa-machi 3-chome, Maebashi-shi, Gunma Prefecture 371-8511 Japan; 8 Department of Breast Surgery, International University of Health and Welfare, 4-3, Kozunomori, Narita-shi, Chiba Prefecture 286-8686 Japan

**Correction**: **Breast Cancer Research (2023) 25:13** https://doi.org/10.1186/s13058-023-01606-7

Following publication of the original article [1], an error was identified in the Discussion section.

The updated conclusion is given below and the changes have been highlighted in bold typeface.

“According to hormone receptor status, we found that ER positivity was associated with decreased PD-L1 expression in Mo/Mφ and mDC (Additional file 1: Figure S2d, h), PgR positivity with increased PD-L1 expression in MDSCs and decreased PD-L1 expression in NK cells (Additional file 1: Figure S3f, j), and AR positivity with increased PD-L1 expression in CD8+ T and decreased PD-L1 expression in Mo/Mφ cells (Additional file 1: Figure S4b, d). PD-L1 expression reflects ongoing (or active) immune responses in addition to immunosuppression via the PD-1/PD-L1 pathway [60–62]. Thus, ER positivity and AR positivity may reflect specific immune response in the breast cancer microenvironment.”

The original article [1] has been corrected.
