# Supplementary material for: Expression of hormone receptors is associated with specific immunological profiles of the breast cancer microenvironment
Source: Breast Cancer Res. 2023 Jan 31;25:13. doi: 10.1186/s13058-023-01606-7 (PMC9887885; doi:10.1186/s13058-023-01606-7)
Supplement: Supplementary file 1 — Additional file 1: Fig. S1. Association of typical biological processes with the expression of hormone receptors estimated using the gene expression profile of breast cancer. (a-f) Summary of results of GSEA performed on the METABRIC datasets is shown. Biological processes positively or inversely correlated with each gene expression are shown in the descending order of the absolute value of the normalized enrichment score (NSE) with absolute values of the log-transformed nominal p-values and FDR q-values. Thresholds of the nominal p-value and FDR q-value were set to < 0.05 and < 0.25, respectively, and the boundaries are shown in the graph by dashed lines. Fig. S2. Programmed death-ligand 1 (PD-L1) positive ratio in each immune cell fraction according to estrogen receptor (ER) status. (a-k) the percentage of PD-L1 positive cells in each immune cell fraction by ER status is shown. A summary of statistics value is shown at left side of figure. A summary of the recalculated statistics values excluding outliers is shown in the lower left of the figure. Fig. S3. Programmed death-ligand 1 PD-L1 positive ratio in each immune cell fraction according to progesterone receptor (PgR) status. (a-k) the percentage of PD-L1 positive cells in each immune cell fraction by PgR status is shown. A summary of statistics value is shown at left side of figure. A summary of the recalculated statistics values excluding outliers is shown in the lower left of the figure. Fig. S4. Programmed death-ligand 1 (PD-L1) positive ratio in each immune cell fraction according to androgen receptor (AR) status. (a-k) the percentage of PD-L1 positive cells in each immune cell fraction by AR status is shown. A summary of statistics value is shown at left side of figure. A summary of the recalculated statistics values excluding outliers is shown in the lower left of the figure. Fig. S5. Reanalysis of Figs. 2–4 using 1% cut-off point for ER and PgR. (a-d) Correlation of hTIL and hPD-L1 with protein expressio [file 13058_2023_1606_MOESM1_ESM.docx]

**Additional file**

**a**

**d**

**b**

**e**

**c**

**f**

**Fig. S1 Association of typical biological processes with the expression of hormone receptors estimated using the gene expression profile of breast cancer.** (a-f) Summary of results of GSEA performed on the METABRIC datasets is shown. Biological processes positively or inversely correlated with each gene expression are shown in the descending order of the absolute value of the normalized enrichment score (NSE) with absolute values of the log-transformed nominal p-values and FDR q-values. Thresholds of the nominal p-value and FDR q-value were set to <0.05 and <0.25, respectively, and the boundaries are shown in the graph by dashed lines.

**a**

**b**

**c**

**d**

**e**

**f**

**g**

**h**

**i**

**j**

**k**

**Fig. S2 Programmed death-ligand 1 (PD-L1) positive ratio in each immune cell fraction according to estrogen receptor (ER) status.** (a-k) the percentage of PD-L1 positive cells in each immune cell fraction by ER status is shown. A summary of statistics value is shown at left side of figure. A summary of the recalculated statistics values excluding outliers is shown in the lower left of the figure.

**a**

**b**

**c**

**d**

**e**

**f**

**g**

**h**

**i**

**j**

**k**

**Fig. S3 Programmed death-ligand 1 PD-L1 positive ratio in each immune cell fraction according to progesterone receptor (PgR) status.** (a-k) the percentage of PD-L1 positive cells in each immune cell fraction by PgR status is shown. A summary of statistics value is shown at left side of figure. A summary of the recalculated statistics values excluding outliers is shown in the lower left of the figure.

**a**

**b**

**c**

**d**

**e**

**f**

**g**

**h**

**i**

**j**

**k**

**Fig. S4 Programmed death-ligand 1 (PD-L1) positive ratio in each immune cell fraction according to androgen receptor (AR) status.** (a-k) the percentage of PD-L1 positive cells in each immune cell fraction by AR status is shown. A summary of statistics value is shown at left side of figure. A summary of the recalculated statistics values excluding outliers is shown in the lower left of the figure.

*p < 0.001**

*p = 0.137**

*p = 0.014**

*p = 0.027**

**a**

**b**

**c**

**d**

*Fisher's exact test

**e**

**f**

**g**

**h**

**Fig. S5 Reanalysis of figures 2–4 using 1% cut-off point for ER and PgR. (a-d) Correlation of hTIL and hPD-L1 with protein expression of ER and PgR were reanalyzed using the cut-off points 1% for ER and PgR.** (e, f) Association between ER / PgR status and subsets of tumor-infiltrating immune cells were reanalyzed using the cut-off points 1% for ER and PgR. (g, h) Recalculated statistics values excluding outliers are shown for ER and PgR status.

| Table S1. Clinical-pathological characteristics by ER status | | | |
| --- | --- | --- | --- |
|  | ER Positive (N=17) | ER Negative (N=28) | *p*-value* |
| Menopausal status |  |  | 0.198 |
| Post | 9.0 (52.9%) | 20.0 (74.1%) |  |
| Pre | 8.0 (47.1%) | 7.0 (25.9%) |  |
| Unknown | 0 | 1 |  |
| Neo-adjuvant thrrapy |  |  | 0.452 |
| present | 4.0 (23.5%) | 4.0 (14.3%) |  |
| absent | 13.0 (76.5%) | 24.0 (85.7%) |  |
| Hystological Type |  |  | 0.057 |
| IDC | 16.0 (94.1%) | 23.0 (82.1%) |  |
| ILC | 1.0 (5.9%) | 0.0 (0.0%) |  |
| Special | 0.0 (0.0%) | 5.0 (17.9%) |  |
| Invasive tumor size |  |  | 0.689 |
| ≥20mm | 14.0 (87.5%) | 22.0 (78.6%) |  |
| < 20mm | 2.0 (12.5%) | 6.0 (21.4%) |  |
| Unknown | 1 | 0 |  |
| Lymph node metastasis |  |  | 0.533 |
| Positive | 9.0 (56.2%) | 12.0 (42.9%) |  |
| Negative | 7.0 (43.8%) | 16.0 (57.1%) |  |
| Unknown | 1 | 0 |  |
| Lymphatic invasion |  |  | 0.344 |
| Positive | 11.0 (68.8%) | 14.0 (50.0%) |  |
| Negative | 5.0 (31.2%) | 14.0 (50.0%) |  |
| Unknown | 1 | 0 |  |
| Vascular invasion |  |  | 0.652 |
| Positive | 3.0 (18.8%) | 3.0 (10.7%) |  |
| Negative | 13.0 (81.2%) | 25.0 (89.3%) |  |
| Unknown | 1 | 0 |  |
| Histological Grade |  |  | 0.001 |
| Grade 3 | 0.0 (0.0%) | 11.0 (45.8%) |  |
| Grade 1 or 2 | 16.0 (100.0%) | 13.0 (54.2%) |  |
| Unknown | 1 | 4 |  |
| PgR status |  |  | <.001 |
| Positive | 7.0 (41.2%) | 0.0 (0.0%) |  |
| Negative | 10.0 (58.8%) | 28.0 (100.0%) |  |
| AR status |  |  | 0.005 |
| High | 14.0 (82.4%) | 10.0 (35.7%) |  |
| Low | 3.0 (17.6%) | 18.0 (64.3%) |  |
| HER2 status |  |  | 0.455 |
| Positive | 2.0 (11.8%) | 6.0 (22.2%) |  |
| Negative | 15.0 (88.2%) | 21.0 (77.8%) |  |
| Unknown | 0 | 1 |  |
| Ki67 RI |  |  | 0.163 |
| ≥ 20% | 10.0 (58.8%) | 23.0 (82.1%) |  |
| < 20% | 7.0 (41.2%) | 5.0 (17.9%) |  |
| *Fisher's exact test | | | |

| Table S2. Clinical-pathological characteristics by PgR status | | | |
| --- | --- | --- | --- |
|  | PgR Positive (N=7) | PgR Negative (N=38) | *p*-value* |
| Menopausal status |  |  | 0.036 |
| Post | 2.0 (28.6%) | 27.0 (73.0%) |  |
| Pre | 5.0 (71.4%) | 10.0 (27.0%) |  |
| Unknown | 0 | 1 |  |
| Neo-adjuvant thrrapy |  |  | 1.000 |
| present | 1.0 (14.3%) | 7.0 (18.4%) |  |
| absent | 6.0 (85.7%) | 31.0 (81.6%) |  |
| Hystological Type |  |  | 0.641 |
| IDC | 7.0 (100.0%) | 32.0 (84.2%) |  |
| ILC | 0.0 (0.0%) | 1.0 (2.6%) |  |
| Special | 0.0 (0.0%) | 5.0 (13.2%) |  |
| Invasive tumor size |  |  | 0.593 |
| ≥20mm | 5.0 (71.4%) | 31.0 (83.8%) |  |
| < 20mm | 2.0 (28.6%) | 6.0 (16.2%) |  |
| Unknown | 0 | 1 |  |
| Lymph node metastasis |  |  | 1.000 |
| Positive | 3.0 (42.9%) | 18.0 (48.6%) |  |
| Negative | 4.0 (57.1%) | 19.0 (51.4%) |  |
| Unknown | 0 | 1 |  |
| Lymphatic invasion |  |  | 1.000 |
| Positive | 4.0 (57.1%) | 21.0 (56.8%) |  |
| Negative | 3.0 (42.9%) | 16.0 (43.2%) |  |
| Unknown | 0 | 1 |  |
| Vascular invasion |  |  | 1.000 |
| Positive | 1.0 (14.3%) | 5.0 (13.5%) |  |
| Negative | 6.0 (85.7%) | 32.0 (86.5%) |  |
| Unknown | 0 | 1 |  |
| Histological Grade |  |  | 0.159 |
| Grade 3 | 0.0 (0.0%) | 11.0 (33.3%) |  |
| Grade 1 or 2 | 7.0 (100.0%) | 22.0 (66.7%) |  |
| Unknown | 0 | 5 |  |
| ER status |  |  | <0.001 |
| Positive | 7.0 (100.0%) | 10.0 (26.3%) |  |
| Negative | 0.0 (0.0%) | 28.0 (73.7%) |  |
| AR status |  |  | 0.422 |
| High | 5.0 (71.4%) | 19.0 (50.0%) |  |
| Low | 2.0 (28.6%) | 19.0 (50.0%) |  |
| HER2 status |  |  | 0.318 |
| Positive | 0.0 (0.0%) | 8.0 (21.6%) |  |
| Negative | 7.0 (100.0%) | 29.0 (78.4%) |  |
| Unknown | 0 | 1 |  |
| Ki67 RI |  |  | 0.362 |
| ≥ 20% | 4.0 (57.1%) | 29.0 (76.3%) |  |
| < 20% | 3.0 (42.9%) | 9.0 (23.7%) |  |
| *Fisher's exact test | | | |

| Table S3. Clinical-pathological characteristics by AR status | | | |
| --- | --- | --- | --- |
|  | AR high (N=24) | AR low (N=21) | *p*-value* |
| Menopausal status |  |  | 1.000 |
| Post | 16.0 (66.7%) | 13.0 (65.0%) |  |
| Pre | 8.0 (33.3%) | 7.0 (35.0%) |  |
| Unknown | 0 | 1 |  |
| Neo-adjuvant thrrapy |  |  | 1.000 |
| present | 4.0 (16.7%) | 4.0 (19.0%) |  |
| absent | 20.0 (83.3%) | 17.0 (81.0%) |  |
| Hystological Type |  |  | 1.000 |
| IDC | 20.0 (83.3%) | 19.0 (90.5%) |  |
| ILC | 1.0 (4.2%) | 0.0 (0.0%) |  |
| Special | 3.0 (12.5%) | 2.0 (9.5%) |  |
| Invasive tumor size |  |  | 0.448 |
| ≥20mm | 20.0 (87.0%) | 16.0 (76.2%) |  |
| < 20mm | 3.0 (13.0%) | 5.0 (23.8%) |  |
| Unknown | 1 | 0 |  |
| Lymph node metastasis |  |  | 0.246 |
| Positive | 13.0 (56.5%) | 8.0 (38.1%) |  |
| Negative | 10.0 (43.5%) | 13.0 (61.9%) |  |
| Unknown | 1 | 0 |  |
| Lymphatic invasion |  |  | 0.127 |
| Positive | 16.0 (69.6%) | 9.0 (42.9%) |  |
| Negative | 7.0 (30.4%) | 12.0 (57.1%) |  |
| Unknown | 1 | 0 |  |
| Vascular invasion |  |  | 1.000 |
| Positive | 3.0 (13.0%) | 3.0 (14.3%) |  |
| Negative | 20.0 (87.0%) | 18.0 (85.7%) |  |
| Unknown | 1 | 0 |  |
| Histological Grade |  |  | 0.001 |
| Grade 3 | 1.0 (4.8%) | 10.0 (52.6%) |  |
| Grade 1 or 2 | 20.0 (95.2%) | 9.0 (47.4%) |  |
| Unknown | 3 | 2 |  |
| ER status |  |  | 0.005 |
| Positive | 14.0 (58.3%) | 3.0 (14.3%) |  |
| Negative | 10.0 (41.7%) | 18.0 (85.7%) |  |
| PgR status |  |  | 0.422 |
| Positive | 5.0 (20.8%) | 2.0 (9.5%) |  |
| Negative | 19.0 (79.2%) | 19.0 (90.5%) |  |
| HER2 status |  |  | 0.710 |
| Positive | 5.0 (20.8%) | 3.0 (15.0%) |  |
| Negative | 19.0 (79.2%) | 17.0 (85.0%) |  |
| Unknown | 0 | 1 |  |
| Ki67 RI |  |  | 0.329 |
| ≥ 20% | 16.0 (66.7%) | 17.0 (81.0%) |  |
| < 20% | 8.0 (33.3%) | 4.0 (19.0%) |  |
| *Fisher's exact test | | | |
